# *Taylorella equigenitalis* infections in Poland – results of current diagnostic investigations

**DOI:** 10.2478/jvetres-2025-0040

**Published:** 2025-08-07

**Authors:** Bernard Wasiński, Jolanta Złotnicka, Maria Kubajka, Martyna Olejarczyk, Krzysztof Szulowski

**Affiliations:** 1 Department of Bacteriology and Bacterial Animal Diseases National Veterinary Research Institute, 24-100 Puławy, Poland

**Keywords:** contagious equine metritis, equine venereal disease, genital infection, horse breeding, *Taylorella equigenitalis*

## Abstract

**Introduction:**

Contagious equine metritis (CEM) is a cosmopolitan infectious, venereal disease of equids caused by the bacterium *Taylorella equigenitalis*. Its frequently asymptomatic course leads to its prevalence sometimes being underestimated, and knowledge of the spread of infections with its causative agent is insufficient. The aim of this study is to summarise and present data on the incidence of *T. equigenitalis* infections in horses in Poland.

**Material and Methods:**

In the years 2018–2023, routine laboratory tests of horses for CEM were carried out. Between 52 and 99 horses were examined annually. Swabs from the external parts of the urogenital system of mares and stallions were investigated. The supplied swabs were inoculated onto microbiological media and then DNA was isolated from each swab for PCR.

**Results:**

In 2018 and 2020, no horses infected with *T. equigenitalis* were found by any method. In other years, from 1 to 3 (1.01–4.41%) infected horses were found. All positive horses were stallions and came from stables located in central Poland.

**Conclusion:**

The detection of infected horses in relatively small groups of tested animals indicates an urgent need to introduce monitoring to estimate the spread of *T. equigenitalis* infections throughout the country. This may facilitate a more precise assessment of the prevalence of *T. equigenitalis* infections in Poland and, consequently, their more effective containment.

## Introduction

*Taylorella equigenitalis* is the aetiological agent of a highly infectious equine venereal disease called contagious equine metritis (CEM). This bacterium (also referred to as the contagious equine metritis organism – CEMO) is a pathogen of equids and does not affect other livestock or people. Shortly after the description of the world’s first case recognised as CEM in the United Kingdom in 1977 (2, 22), equine infections caused by *T. equigenitalis* were confirmed in France (16), Germany (1), the USA (20), Japan (10), Australia (7), South Africa (14) and numerous other countries (9, 26). Two biotypes of CEMO have been identified, the first of which is susceptible to and the second resistant to streptomycin (15).

Stallions are asymptomatic carriers and probably primary reservoirs of *T. equigenitalis*. The bacterium persistently colonises primarily the external parts of the urogenital tract of stallions, which appear to be unable to clear infection without treatment (21). The transmission of CEMO occurs mainly during mating, while a lower risk is associated with artificial insemination or embryo transfer. Nevertheless, owing to the increased global trade in semen for artificial insemination in the equine breeding industry, biosecurity and import/export regulations have taken on increased significance (18). The European Union requires rigorous testing of breeding stallions before the semen collection season (4), which effectively prevents the transmission of CEMO through fresh and frozen semen. However, under current EU regulations, stallions used for natural service within a single member state are not subject to mandatory CEM testing, unless national legislation requires it. Vertical transmission of infection is also possible. Foals may become infected in the uterus, during parturition or later through contact with an environment contaminated with amniotic fluid, remnants of the placenta or foetal membranes. It is also possible for CEMO to be transferred from stallions to foals. This is very seldom through direct contact and more often through fomites, in particular animal care equipment (combs, cleaning sponges, *etc*.). Foals infected at foaling may become long-term subclinical carriers (17). Beyond the foal stage, younger horses may be more susceptible to high-intensity CEMO infections. Notably, infection appears to be more likely in geldings and maiden mares compared to breeding mares (18). These findings contrast with earlier assumptions that horses involved in breeding were at the highest risk of CEMO infection.

Approximately 60–70% of mares infected with *T. equigenitalis* do not exhibit any clinical signs and at least this many are able clear the infection without treatment within a few months, unlike stallions (12, 13, 18). Although clinical recovery occurs in the majority of cases, some mares become carriers. When signs do appear, it is usually 1–3 days after mating. They most often include slight to copious mucopurulent vaginal discharges. They are a consequence of variable endometritis, cervicitis and/or vaginitis. Another frequent symptom, observed often in primary cases of CEM, is a mare returning to oestrus prematurely after being bred (17, 24). Temporary infertility is not uncommon in the acute form of the disease, whereas abortions are extremely rare.

Antimicrobial and disinfectant therapy for both mares and stallions typically lasts for 5–7 days. It does not always produce satisfactory results, and repeated applications may be required. Persistent carrier status of a horse is a constant threat to other horses and incurs substantial expenses for its owner. The financial losses associated with CEM are not limited to temporary infertility, a lower number of achieved pregnancies, sporadic abortions or the elimination of semen collected from infected stallions. They also arise from substantial indirect expenses associated with outbreak management and treatment, quarantine and surveillance measures, and international trade and movement restrictions. The cost of eliminating the first recognised CEM outbreak in the United Kingdom (which infected approximately 200 mares and over 20 stallions) was estimated at $20–30 million. In the United States the first identified outbreak occurred in 1978 in Kentucky and the estimated cost of eradicating CEMO was $13.5 million (11).

Effective prevention of CEMO transmission is largely based on laboratory bacteriological and molecular testing of horses utilised for reproduction. Laboratory tests are most often performed on samples from breeding horses transferred to other countries. The serious economic losses to the horse-breeding industry caused by CEM have led many countries to implement monitoring of *T. equigenitalis* infections, which allows for more effective control of this disease. Infections with CEMO have not been systematically monitored in Poland until now. However, some breeders require annual pre-mating tests for their breeding horses, and tests of exported breeding horses are conducted. The aims of this article are to summarise the results of routine diagnostic tests for *T. equigenitalis* infections performed in Poland in the years 2018–2023 and to present the conclusions resulting from them.

## Material and Methods

### Animals

The number of horses examined in one year in the study duration from 2018 to 2023 ranged from 52 to 99 (Table 1). Among the horses tested each year, the majority were stallions (68.75–88.46%). The horses examined came from both large state-owned herds and smaller private stables located mostly in central and southern Poland. The samples for the studies were mainly provided by owners intending to sell their horses or breed them. Consequently, those horses were not reported to be exhibiting any signs of illness.

**Table 1.** Comparison of the number of horses tested for *Taylorella equigenitalis* and showing positive results in particular years

Year	Number of horses tested	Number/% of tested		Number/% of positive horses	Sex of positive horses
		mares	stallions		
2018	64	20/31.25	44/68.75		
2019	68	9/13.24	59/86.76	1/1.47	♂
2020	52	6/11.54	46/88.46		
2021	99	19/19.20	80/80.80	1/1.01	♂
2022	80	14/17.50	66/82.50	1/1.25	♂
2023	68	12/17.65	56/82.35	3/4.41	3 × ♂

### Samples collection and transport

Swabs from the external genital tracts of mares or the external urogenital tracts of stallions were collected and sent for testing. Swabs were collected twice every seven days from each horse subjected to testing. Three swabs were collected from each mare from the following sites: the vaginal vestibule, the clitoral fossa and the periclitoral sinuses. In stallions, swabs were taken from the penile sheath (prepuce), the urethra and the fossa glandis. Directly after collection, all swabs were submerged in Amies charcoal transport medium and transported under cool conditions to the laboratory of the National Veterinary Research Institute in Puławy, Poland, where they were examined. Samples were cultured within 48 h of collection.

### Isolation of *T. equigenitalis*

Culture was performed according to recommended procedures (24) with the use of three media. The first was chocolate agar containing clindamycin (5 μg/mL), trimethoprim (1 μg/mL) and amphotericin B (5 μg/mL). The composition of inhibitors included in this medium allowed the growth of streptomycin-resistant and -susceptible biotypes and inhibited the growth of commensal bacteria and fungi. The second was chocolate blood agar containing streptomycin sulphate (200 μg/mL) for differentiation of streptomycin-resistant or -susceptible biotypes. The third was chocolate blood agar without the inhibitors which could prevent the isolation of some strains of *T. equigenitalis*. Each swab was inoculated on the first and second media and then tested by real time PCR. The cultures were incubated for at least 7 d at 37°C in 7% (v/v) CO_2_ in air. As part of the bacteriological investigation, the colonies suspected of being *T. equigenitalis* found on the first and/or the second medium were re-inoculated on the third medium without inhibitors. They were tested for the production of oxidase and catalase, typed serologically and subjected to real-time PCR.

### Serological typing

A latex agglutination test (Monotayl; Bionor Laboratories, Skein, Norway) was used for serological typing of isolates suspected of being *T. equigenitalis*. The test was performed according to the manufacturer’s instructions.

### DNA extraction and real-time PCR

As previously mentioned, DNA extraction was performed from each swab tested and from suspensions prepared from colonies suspected of being *T. equigenitalis* as a confirmatory step. The extraction was performed using a DNeasy Blood and Tissue kit (Qiagen, Venlo, the Netherlands) according to the manufacturer’s instructions. The real time PCR was performed as previously described by Wakeley *et al*. (23). Briefly, oligonucleotide primers with the sequences 5′-CCGCGTGTGCGATTGA-3′ and 5′-TTTGCCGGTGCTTATTCTTCA-3′ at 400 nM concentration, and a probe with the sequence 5′-FAM-AAAGGTTTGTGTTAATACCATGGACTGCTGAG-BHQ1-3′ at 100 nM were used. The thermal profile of the real-time PCR was set as 95°C for 5 min, 42 cycles of 94°C for 15 s and 60°C for 60 s. Positive and negative controls were added to each run. A positive test had a cycle threshold value ≤35.

No growth of suspected *T. equigenitalis* colonies after 7 d of incubation was the criterion for tested samples to be considered CEMO negative. A horse showing negative results in two consecutive bacteriological tests was certified CEMO negative. Horses with a positive result (isolation of *T. equigenitalis*) in at least one test were considered infected, and their owners were advised to conduct further testing on their animals.

The real-time PCR was used as an additional test, and the result of the bacteriological test was essential for determining the final result. In the case of a positive result only from the real-time PCR (with a negative bacteriological test result), the horse was suspected of carrying an infection and the owner was informed.

## Results

**Fig. 1.**
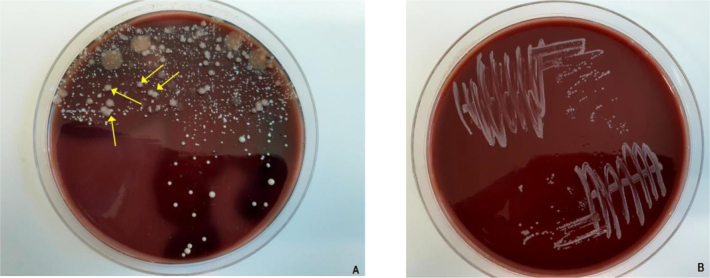
Growth of *Taylorella equigenitalis* on chocolate agar. A) colonies (indicated by arrows) among mixed cultures of bacteria grown in direct inoculations from urogenital swabs of stallions onto agar. B) isolated cultures

During the six-year investigation, 6 cases of isolation of *T. equigenitalis* were found (Table 1). All strains were isolated from stallions. No infected animals were found in 2018 or 2020, but those were years in which relatively fewer horses were tested. The strains isolated (Fig. 1) in 2019, 2021 and 2022 were streptomycin susceptible. In 2023 three horses infected with streptomycin-resistant strains were found (Table 2). All three of those horses were kept in one stable.

**Table 2.** Comparison of the age and breed of stallions identified as infected with *Taylorella equigenitalis* and of the laboratory results of their urogenital swabs

Year of examination	Age (years)	Breed	Culture (isolation)	Streptomycin susceptibility/resistance	Serological identification (latex agglutination)	Oxidase production	Catalase production	Real-time PCR
2019	5	Arabian	+	susceptible	*T. equigenitalis*	+	+	+
2021	19	Malopolski	+	susceptible	*T. equigenitalis*	+	+	+
2022	20	Malopolski	+	susceptible	*T. equigenitalis*	+	+	+
2023	4	Anglo European Sport Horse (AES)	+	resistant	*T. equigenitalis*	+	+	+
2023	4	Westphalian	+	resistant	*T. equigenitalis*	+	+	+
2023	4	Sella Italiano	+	resistant	*T. equigenitalis*	+	+	+

**Fig. 2.**
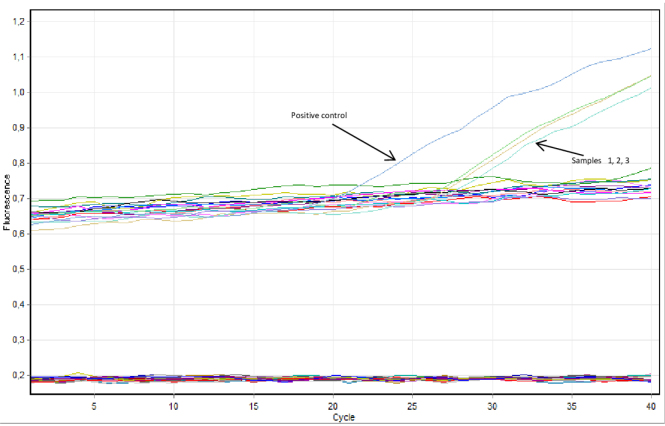
Real-time PCR amplification curves showing positive detections of *Taylorella equigenitalis* DNA in urogenital swabs from stallions

The affiliation of each isolated strain to the *T. equigenitalis* species was confirmed by latex agglutination tests and real time PCR (Fig. 2). All six cases of positivity for CEMO shown by isolation were further confirmed by the direct detection of *T. equigenitalis* DNA in tested swabs. The horses from which *T. equigenitalis* was isolated came from stables located in central Poland.

In addition, in 2019 a positive test result was found for one stallion after the first sampling using the real-time PCR method only, and bacteriological examination did not detect the presence of *T. equigenitalis*. The horse’s owner declined to have the horse sampled a second time after being informed of this result. This particular test remained inconclusive; therefore, this horse was not included in the group of tested animals presented in Tables 1 and 2.

## Discussion

In 2004, CEM was diagnosed in Poland for the first time (25). During further investigations conducted between 2007 and 2009, in which a total of 212 horses were examined, *T. equigenitalis* was isolated from two Polish stallions (25). Currently and as they were at the time of that research, diagnostic laboratory tests for CEMO are performed in Poland in a single state laboratory (the National Veterinary Research Institute) and usually cover less than 100 horses annually. The number of horses with positive results varies between one and three per year, although there are often years when no positive results are found. The results from the last six years presented here seem to confirm this tendency.

In certain instances, the laboratory was unable to obtain information regarding the age and/or breed of the tested animals, which is a limitation of the present work. Nonetheless, all horses identified as CEMO-positive had accessible age and breed data. Further limitations of this study included the low number of animals tested and some inconsistency in sampling resulting from sample selection having been solely at the discretion of the horse owners. Moreover, the majority of tested horses came from studs located mostly in central and southern Poland, and only a few samples were collected from animals kept in the northern part of the country, implying that the results may not be representative of the country-wide picture. It seems, however, that even based on the rough results obtained, it is reasonable to conclude that the prevalence of CEMO infections in equine populations in Poland is underestimated. The detection of infected horses among a relatively small number of animals tested may indicate a significant spread of CEMO in the domestic horse population. However, to obtain more reliable data in this area, it would be necessary to implement broader monitoring.

A similar problem appears to occur in various European countries as well as on other continents (see Introduction), especially with regard to horses used for pasture breeding. However, a recent report indicates that the incidence of *T. equigenitalis* DNA (found using quantitative PCR) in Icelandic brood mares kept in Southern Germany and Austria was lower (2.2%) than among maiden mares (9.0%) and significantly lower than among geldings (36.2%) (18). Moreover, the threshold cycle values observed during this investigation were significantly lower for samples from geldings than for those from brood and maiden mares (18).

The usually mild and often asymptomatic courses of infections caused by CEMO mean that these infections are often underestimated, and broader actions to eradicate them (especially well-organised monitoring) are often neglected or postponed. The insidious nature of CEM means that the economic losses caused by this disease may extend over longer periods of time, especially when infections are already widespread in a given area.

Despite the articles pertaining to the occurrence of *T. equigenitalis* in horses being numerous, results of studies covering entire countries are rare. The majority of reports contain data from selected regions of multiple countries or sometimes from selected stables/studs or from a group of independent stables located in different parts of a given country, or the reports present the results of screening selected horse breeds.

Information about infections caused by CEMO has come from most of the countries neighbouring Poland, such as Austria, the Czech Republic, Denmark (sharing a maritime border), Germany, Slovakia and Sweden (1, 3, 6, 8, 18, 19). There is no information from the countries bordering it to the east. Nonetheless, even the limited available information supports the assumption that there are favourable conditions for the spread of the disease, given the currently developing cross-border trade in horses. The reported incidence of CEMO infections in European countries frequently reflects the depth and breadth of routine laboratory diagnostic tests for CEM in those countries. It appears that international transparency concerning the occurrence of CEM in individual countries and reliable information on the measures taken to prevent its spread would be helpful to individual horse breeders and the whole international equine trade. This would ultimately effectively help prevent the spread of CEM. Controlling venereally transmitted diseases is important for any breeding programme and for successful exports (17). The fundamental measures to prevent the spread of CEMO infections entail introducing solely horses with negative laboratory results into the country, and only permitting animals free from *T. equigenitalis* to breed. Another important part of eradicating CEM is the widest possible implementation of laboratory tests for monitoring. In countries or regions with a poorly recognised epizootic CEM situation, the appropriate selection of horses for sampling is especially important. Firstly, the tested groups should include an adequate number of horses used for pasture breeding, as the risk of CEMO infections seems to be particularly high among these animals. Secondly, taking into account the recently published results from Germany and Austria (18), the occurrence of CEMO infections in horses not used for breeding should also be carefully investigated through the sampling of such animals. We believe that the pilot monitoring of *T. equigenitalis* infections in stallions initiated in Poland in 2024 will improve our understanding of the epizootic situation and will allow for more effective control of CEM. Nevertheless, it should merely serve as a primer for more extensive monitoring.

## Conclusion

The results of the presented investigations confirm the occurrence of CEMO infections in horses in Poland. The detection of horses infected with *T. equigenitalis* in selected years (with an incidence of 1–4.4%) in relatively small groups of examined animals suggests the urgent need for broader monitoring studies throughout the country. The results of such monitoring would allow for a more precise determination of the spread of *T. equigenitalis* infections in the Polish horse population and more effective control of CEM on a national scale.
